# Exacerbating effects of single-dose acute ethanol exposure on neuroinflammation and amelioration by GPR110 (ADGRF1) activation

**DOI:** 10.1186/s12974-023-02868-w

**Published:** 2023-08-14

**Authors:** Sharmistha Banerjee, Taeyeop Park, Yoo Sun Kim, Hee-Yong Kim

**Affiliations:** 1 Laboratory of Molecular Signaling, National Institute on Alcohol Abuse and Alcoholism, 5625 Fishers Lane, Rockville, MD 20852 USA; 2 National Institutes of Health, 5625 Fishers Lane, Rm. 3N-07, Bethesda, MD 20892-9410 USA

**Keywords:** Adenylyl cyclase, Phosphodiesterase, cAMP, Cytokines, Gavage, GPCR, NLRP3 inflammasome, Lipopolysaccharide, Macrophages, Microglia, Synaptamide

## Abstract

**Background:**

Neuroinflammation is a widely studied phenomenon underlying various neurodegenerative diseases. Earlier study demonstrated that pharmacological activation of GPR110 in both central and peripheral immune cells cooperatively ameliorates neuroinflammation caused by systemic lipopolysaccharide (LPS) administration. Ethanol consumption has been associated with exacerbation of neurodegenerative and systemic inflammatory conditions. The goal of this study is to determine the effects of single-dose acute ethanol exposure and GPR110 activation on the neuro-inflammation mechanisms**.**

**Methods:**

For in vivo studies, GPR110 wild type (WT) and knockout (KO) mice at 10–12 weeks of age were given an oral gavage of ethanol (3 g/kg) or maltose (5.4 g/kg) at 1–4 h prior to the injection of LPS (1 mg/kg, i.p.) followed by the GPR110 ligand, synaptamide (5 mg/kg). After 2–24 h, brains were collected for the analysis of gene expression by RT-PCR or protein expression by western blotting and enzyme-linked immunosorbent assay (ELISA). Microglial activation was assessed by western blotting and immunohistochemistry. For in vitro studies, microglia and peritoneal macrophages were isolated from adult WT mice and treated with 25 mM ethanol for 4 h and then with LPS (100 ng/ml) followed by 10 nM synaptamide for 2 h for gene expression and 12 h for protein analysis.

**Results:**

Single-dose exposure to ethanol by gavage before LPS injection upregulated pro-inflammatory cytokine expression in the brain and plasma. The LPS-induced Iba-1 expression in the brain was significantly higher after ethanol pretreatment in both WT and GPR110KO mice. GPR110 ligand decreased the mRNA and/or protein expression of these cytokines and Iba-1 in the WT but not in GPR110KO mice. In the isolated microglia and peritoneal macrophages, ethanol also exacerbated the LPS-induced expression of pro-inflammatory cytokines which was mitigated at least partially by synaptamide. The expression of an inflammasome marker NLRP3 upregulated by LPS was further elevated with prior exposure to ethanol, especially in the brains of GPR110KO mice. Both ethanol and LPS reduced adenylate cyclase 8 mRNA expression which was reversed by the activation of GPR110. PDE4B expression at both mRNA and protein level in the brain increased after ethanol and LPS treatment while synaptamide suppressed its expression in a GPR110-dependent manner.

**Conclusion:**

Single-dose ethanol exposure exacerbated LPS-induced inflammatory responses. The GPR110 ligand synaptamide ameliorated this effect of ethanol by counteracting on the cAMP system, the common target for synaptamide and ethanol, and by regulating NLRP3 inflammasome.

**Supplementary Information:**

The online version contains supplementary material available at 10.1186/s12974-023-02868-w.

## Introduction

Neuroinflammation is regarded as one of the early associated hallmark phenomena of several neurodegenerative diseases [1, 2]. Systemic administration of lipopolysaccharide (LPS) is known to cause upregulation of inflammatory cytokines in the brain and thus has been used as model to induce neuroinflammation and study neuroinflammation-associated neurodegenerative diseases [3]. The binding of LPS to toll-like receptor 4 (TLR4) results in the activation of transcription factors that upregulate pro-inflammatory cytokines like IL-1β, IL-6 and TNF-α in the periphery. These cytokines can reach the brain by crossing the blood–brain barrier (BBB) through the systemic circulation and activate microglia, the resident immune cells of the CNS that play a major role in neuro-inflammation.

Ethanol consumption has been associated with systemic and organ-specific inflammatory conditions. For example, prior binge ethanol exposure was shown to potentiate the subsequent microglial responses to the second binge in a rat model of alcohol-induced neurodegeneration with upregulated TNF-α and Iba-1 [4]. The decrease in cAMP appears to play an integral role in the pathophysiologic effect of ethanol [5, 6]. Selective inhibitors of PDE4, a cAMP-degrading phosphodiesterase isoform, have been shown to reduce ethanol-induced liver injury [7] or binge alcohol drinking behavior in mice [8, 9]. In addition, chronic ethanol exposure was shown to significantly increase the LPS-inducible expression of PDE4B, enhancing proinflammatory cytokine expression in monocytes and macrophages [10]. LPS-induced inflammation has been shown to be affected by acute and chronic ethanol exposure in an opposite direction. While alcohol pretreatment for 24 h or daily binge for 3 days was shown to induce hypo-responsiveness to LPS, sensitization to LPS has been demonstrated after prolonged ethanol exposure in cultured human monocytes, mouse macrophages or in vivo [11, 12]. In a separate study, exposure to a single or ten daily doses of ethanol was shown to potentiate LPS-induced increase in some proinflammation mediators in mouse serum, liver or brain when LPS treatment was given at 24 h after the last ethanol exposure to avoid the acute effect of ethanol [13].

The opposite effects of acute and chronic ethanol exposure in LPS-induced inflammation found in human monocytes have been linked to the differential regulation of IRAK-M (IL-1R-associated kinase-monocyte), a negative regulator of IRAK-1. Acute ethanol increases IRAK-M, resulting in reduced NFκB DNA binding and expression of proinflammatory cytokines. In contrast, chronic ethanol treatment decreases IRAK-M, thereby increasing NFκB DNA binding and TNF-α production [12]. It has been also reported that chronic alcohol consumption increases TNF-α in isolated mouse Kupffer cells and RAW 264.7 macrophages through the upregulation of micro-RNA155 [14]. Nevertheless, effects of single-dose ethanol exposure on LPS-induced perturbation of the cAMP system in the context of neuro-inflammation have not been demonstrated.

G-protein coupled receptors (GPCRs) including a few adhesion GPCRs (aGPCR), often play a crucial role in controlling the immune system like transcription or migration of immune cells and super-oxide production through their specific G-protein subunits [15–18]. GPR110, an aGPCR, is the target receptor of *N*-docosahexaenoylethanolamine (synaptamide), an endogenous metabolite of docosahexaenoic acid (DHA) [19]. Recently, synaptamide has been shown to alleviate LPS-induced neuroinflammation through upregulating the cAMP system [20] in a GPR110-dependent manner [21]. As the cAMP system is targeted by both ethanol and GPR110 ligands, two signaling pathways may interact to influence neuroinflammation. In this study, we examined the effects of ethanol and GPR110 activation on LPS-induced neuroinflammation in vivo using a single dose ethanol exposure model and in vitro in cultured microglia and macrophages. We found that single-dose ethanol gavage acutely affects the cAMP system and NLRP3 (NOD-like receptor family pyrin domain containing 3) inflammasome assembly, exacerbating neuroinflammation induced by LPS. Our study also revealed that interaction of GPR110 with ethanol on these targets ameliorates exacerbating effects of ethanol on neuroinflammation.

## Materials and methods

### Chemicals and antibodies

Ethanol and lipopolysaccharides (*Escherichia coli* 055:B5) were purchased from Sigma-Aldrich (St. Louis, MO, USA). Dulbecco’s modified Eagle medium (DMEM) was purchased from American Type Culture Collection (ATCC). Antibodies GAPDH (Rabbit mAb, cat# 2118S), IBA-1(Rabbit mAb, cat# 17198S), NLRP3 (Rabbit mAb, cat# 15101S) and IL-1β (Mouse mAb, cat# 12242S) were bought from Cell Signaling technology and used at (1:1000) dilution as per the manufacturer’s instructions. PDE4B (Rabbit mAb, cat# ab170939) antibody was obtained from Abcam. Secondary antibodies were obtained from Sigma Aldrich. Fetal bovine serum and antibiotics were purchased from Invitrogen (Carlsbad, CA, USA). Primers were obtained from IDT (Coralville, IA, USA).

### Animals

C57BL/6J strain mice were purchased from Charles River Laboratories (Portage, MI, USA). GPR110 (Adgrf1) knock out mice were generated by the Knockout Mouse Project (KOMP) Repository (MMRRC_046507-UCD) on C57BL/6 background. All the experimental procedure involving animals were carried out according to the guiding principles for care and use of animals approved by the National Institute on Alcohol Abuse and Alcoholism (LMS-HK-13).

To determine the desirable time interval between ethanol treatment and LPS injection for testing the effect of ethanol on LPS-induced inflammatory responses, mice of 8–10 weeks of age were first administered ethanol at a dose of 3 g/kg via oral gavage and then injected intraperitoneally with LPS (1 mg/kg) at 1, 4 or 6 h after the ethanol gavage. Control animals were administered maltose at a dose of 5.4 g/kg instead of ethanol. At 2 h after LPS injection, mice were anesthetized with isoflurane and transcardially perfused with chilled PBS and the brain cerebrum was collected for RNA isolation and western blotting. For performing immunostaining, perfusion was carried out with chilled PBS containing 4% paraformaldehyde. When needed, blood was collected by cardiac puncture in a vial coated with anticoagulant after anesthesia before performing perfusion. To test the role of GPR110 activation, WT and GPR110 KO mice were injected with 5 mg/kg of synaptamide following the LPS injection at 4 h after ethanol treatment. The blood ethanol level was measured by Ethanol assay kit (Sigma Aldrich, cat# MAK076).

### Isolation and culture of microglia

Primary microglial cells were isolated from the brains of 8- to 10-week-old mice by magnetic separation as described earlier [20]. Mice were transcardially transfused with cold PBS after anesthesia and brains were collected. The collected brain tissues were sliced and then dissociated with MACS dissociator according to the manufacturer’s protocol. The dissociated brain tissues were filtered through MACS Smart Strainer (70 μm) and centrifuged at 300×*g* for 10 min. The debris was removed, and the pellets were suspended in 90 μL of PBS buffer containing 0.5% BSA followed by the incubation with 10 μL of CD11b microbeads per 10^7^ total cells at 4 °C for 15 min. The cells were washed with 1 mL of cold PBS buffer and then centrifuged at 300×*g* for 5 min. The cell pellet containing beads was resuspended in 500 μL of PBS buffer and applied to LS column (Miltenyi Biotec, City, State, USA) prepared by rinsing with 3 mL of PBS in the magnetic field. The microglial cells were captured on the beads in the column while non-target cells passed through the columns. Upon removal of the column from the magnetic separator, 5 mL of the PBS buffer was added to the LS column to elute the microglial cells from the beads by immediate flushing with a plunger into the columns.

### Isolation of peritoneal macrophage

For isolation of peritoneal macrophages, mice at 8–10 weeks of age were euthanized by CO_2_ asphyxiation. Then 10 mL of PBS with 2% serum, penicillin and EDTA was injected into the peritoneal cavity. Peritoneal lavage was collected through syringe and centrifuged at 1500 rpm for 15 min. The cell pellets were resuspended in DMEM containing 10% FBS and 1% penicillin/streptomycin and then incubated at 37 °C for 24 h._._ Cells were washed with PBS to remove non-adherent cells before treatment.

### Immunostaining

Mice were anesthetized using isoflurane and then trans-cardially transfused with 0.1 M phosphate buffer (pH 7.4) and 4% paraformaldehyde (wt/vol), and the brains were removed. The brain tissues were fixed overnight at 4% paraformaldehyde solution, submerged in 30% sucrose solution at 4 °C, embedded with OCT medium (Tissue-Tek, cat#4583) and stored at − 80 °C. Brains were sectioned sagittally starting from the mid-cerebrum and immune-stained for Iba-1. After incubating with anti- Iba-1 (Wako, cat# 019-19741) at 4°C overnight, the sections were treated with biotin-SP-conjugated goat anti-rabbit IgG (H+L) (Jackson ImmunoResearch labs, INC, Code# 111-065-003, PA, USA) for 1h, and with ABC solution prepared from VECTASTAIN Elite ABC Kit (Vector laboratories, Inc; cat# PK-6100, CA, USA) for 30 min followed by 3,3′-diaminobenzidine (DAB) substrate solution (Vector laboratories, Inc; cat# SK-4100) for visualizationn.

### RNA isolation and quantitative RT-PCR

RNA was extracted using Trizol (Ambion, cat # 15596018) and reverse transcribed to cDNA with reverse transcription kit following manufacturer’s protocol (Applied Biosystems cat# 4368814, CA, USA). The expression of mRNA was measured with SYBR green-based RT-PCR. The relative expression of mRNA was calculated after normalization to GAPDH mRNA which was used as an internal control. Samples were analyzed in triplicates using the QuantStudio 3 Real-Time PCR system (Applied Biosystems by Thermo Fisher Scientific). Primer sequences are indicated below. All data are presented as the fold change compared to the maltose control group (MAL):

**Table:** 

NLRP3	Forward	GAGCCTACAGTTGGGTGAAA
	Reverse	CCTACCAGGAAATCTCGAAGAC
IL-1β	Forward	CCTCACAAGCAGAGCACAA
	Reverse	CCAGCCCATACTTTAGGAAGAC
IL6	Forward	GTCTGTAGCTCATTCTGCTCTG
	Reverse	GAAGGCAACTGGATGGAAGT
TNF	Forward	ACGTCGTAGCAAACCACCAA
	Reverse	AAGGTACAACCCATCGGCTG
GAPDH	Forward	AACAGCAACTCCCACTCTTC
	Reverse	CCTGTTGCTGTAGCCGTATT
ADCY1	Forward	TCTGGTCTGGGTGCATAAAG
	Reverse	CATGTGGAGTTACCACCTACTC
ADCY4	Forward	CACCATGGTGGAATTTGCAGTGGC
	Reverse	GAGGATCTTCGAAGAGGGGAGCTC
ADCY5	Forward	CAATACAGTGAATGTGGCCAGCCG
	Reverse	CAGCAAAGGCAGAAGTTGCTTCTG
ADCY7	Forward	GCACGTGCACATCGGAGTCTTGGT
Reverse	Reverse	CTTGAAACTTGGCAGTGTCTGTAC
ADCY8	Forward	CGCATCTACATCCATCGCTAT
	Reverse	GGTCGAATCTGGCAAAGAGTT
PDE4A	Forward	TCTCCTGGCTCCACATGATA
	Reverse	CTGTCTCCTGCTTCAAACTCTC
PDE4B	Forward	GAGCTACACAGCACCTGTTAT
	Reverse	GGAAGAGAGGGAAGTGTTAGTG
PDE4C	Forward	CACAGCCTCGATGGAGAAAT
	Reverse	GTCTTCCAAGGTGTCCAGAAG
PDE4D	Forward	CCTACTCAGCCATCTGCTTAC
	Reverse	GGGATGTGAAGCCACTTGTA
GPR110	Forward	CCAAGAGAAGCCAAACCTCC
	Reverse	TTCGATAAGCCAGCAGGATG

### Western blotting

Proteins were extracted from brain tissue using 1× Lysis buffer (Cell Signaling Technology, City, State, Country). Protein concentration was measured using BCA reagent, and 20 µg of protein from the cell lysate was separated using SDS-PAGE. The proteins were then electroblotted on to PVDF membrane for 90 min at 100 V at 4 °C. The PVDF membranes were blocked with 5% BSA with TBS-T (20 mM Tris–HCl, pH 7.5, 50 mM NaCl, 0.1% Tween 20) for 60 min at room temperature. After blocking, the membranes were incubated with the respective primary antibodies in 5% BSA overnight at 4 °C: NLRP3 (1:1000), Iba-1 (1:1000), IL-1β (1:1000) and PDE4B (1:1000). The membranes were washed in TBS and then incubated with anti-rabbit or anti-mouse IgG–horseradish peroxidase for 60 min. The proteins were then visualized by chemiluminescence using Azure imaging system (Dublin, California). The image data were processed using Image J software. (Molecular Devices, Sunnyvale, CA, USA). For stripping, the membranes were washed with TBS-T followed by incubation in stripping buffer for 20 min. The membranes were then washed and re-blocked for 30 min with 5% BSA with TBS-T (20 mM Tris–HCl, pH 7.5, 50 mM NaCl, 0.1% Tween 20) and incubated with primary antibody after washing. The same procedure was followed as mentioned above for secondary antibody incubation and visualization.

### ELISA assay

Supernatants were collected after cells were treated with ethanol, LPS and/or synaptamide, and assayed using sandwich ELISA for cytokine production using ELISA kits (Invitrogen Life Technologies, Frederick, MD, USA), according to the manufacturer’s instructions.

### Statistical analysis

The statistical analysis was performed using Student’s *t*-test and a one-way ANOVA followed by Tukey’s post hoc test for multiple comparisons. All results are expressed as mean ± SEM. The mean differences were considered statistically significant when *p* < 0.05.

## Results

### Single-dose ethanol exposure potentiates pro-inflammatory responses induced by LPS

**Fig. 1:**
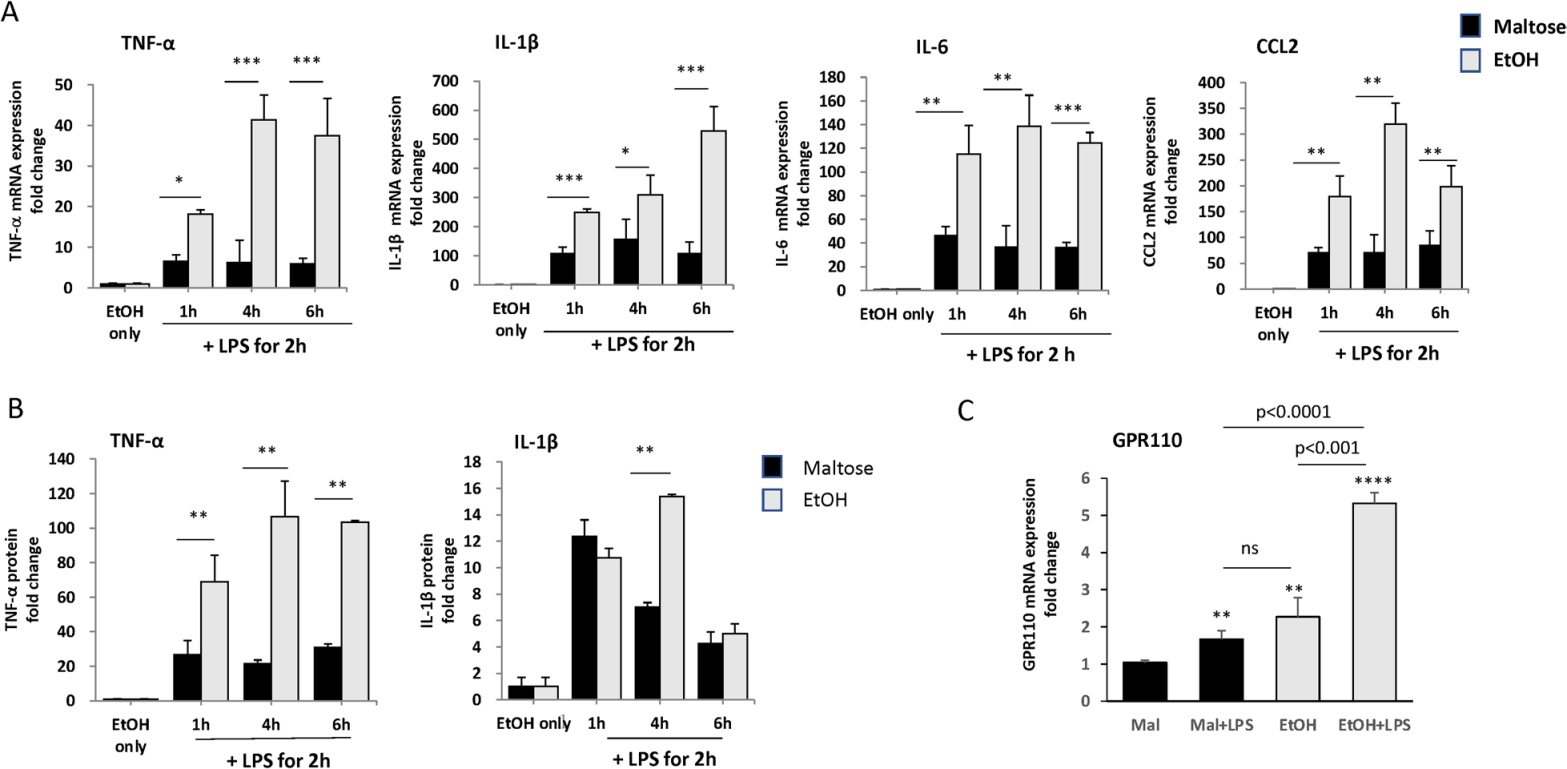
Elevation of LPS-induced inflammatory responses and GPR110 expression in brain after single ethanol exposure. Mice were given ethanol (3 g/kg) or maltose (5.4 g/kg) through oral gavage and LPS (1 mg/kg, i.p.) was injected at 1, 4 and 6 h following ethanol gavage. Brains were collected at 2 h after LPS injection for determining mRNA expression of pro-inflammatory cytokines TNF-α, IL-1β, IL-6 and CCL2 (**A**). The level of pro-inflammatory cytokine TNF-α and IL-1β in blood was measured by ELISA at 2 h after LPS injection made at 1, 4 and 6 h of pretreatment with ethanol gavage (**B**). The transcript level of GPR110 in the brain was quantified after 4 h of ethanol gavage followed by LPS administration for 2 h, in comparison to the group treated with maltose, ethanol or LPS alone (**C**). Values are presented as mean ± SEM (*n* = 3), representing two independent experiments. ns, the difference of means is not statistically significant. *p < 0.05; **p < 0.01; ***p < 0.001; ****p < 0.0001 vs. Maltose group

We first examined whether single-dose ethanol exposure affects LPS-induced inflammatory responses (Fig. 1). When mice were given an oral gavage of ethanol at a dose of 3 g/kg for 1, 4 and 6 h prior to the LPS injection (1 mg/kg, i.p.), ethanol significantly potentiated the mRNA expression of proinflammatory cytokines, TNF-α, IL-1β, IL-6 and CCL2, compared to the maltose-treated controls at all time points examined (Fig. 1A). Ethanol treatment for 4 and 6 h prior to the LPS injection produced the highest increases in the expression of these proinflammatory mediators. The protein level of TNF-α and IL-1β in blood was maximum when ethanol was administered 4 h prior to the LPS injection (Fig. 1B). The single-dose ethanol exposure alone in the absence of LPS did not affect the pro-inflammatory responses in the brain and blood. These results indicated that single ethanol gavage exacerbates LPS-induced neuroinflammation as well as systemic inflammatory responses in vivo. Since the ethanol-enhanced inflammatory response peaked at 4–6 h of ethanol pre-treatment, a single ethanol gavage at 4 h prior to the LPS injection was used in subsequent experiments. The blood ethanol concentration (BAC) for WT mice was 240 ± 21 mg/dL at 1 h and 90 ± 18 mg/dL at 4 h after ethanol gavage. The similar level of BAC was observed for KO mice with 240 ± 23 mg/dL at 1 h and 104 ± 71 mg/dL at 4 h after ethanol gavage.

The expression of GPR110 in the brain has been shown to be high during development, but diminished in the adult stage [19]. Nevertheless, the expression of GPR110 in the adult mouse brain can increase in response to LPS [21] or after traumatic brain injury [22]. We found that GPR110 mRNA also significantly increased in the brain at 4 h after single ethanol gavage, and subsequent LPS injection led to a further increase in the GPR110 expression (Fig. 1C).

### GPR110 activation by synaptamide ameliorates LPS-induced proinflammatory cytokine expression exacerbated by single dose administration of ethanol

**Fig. 2:**
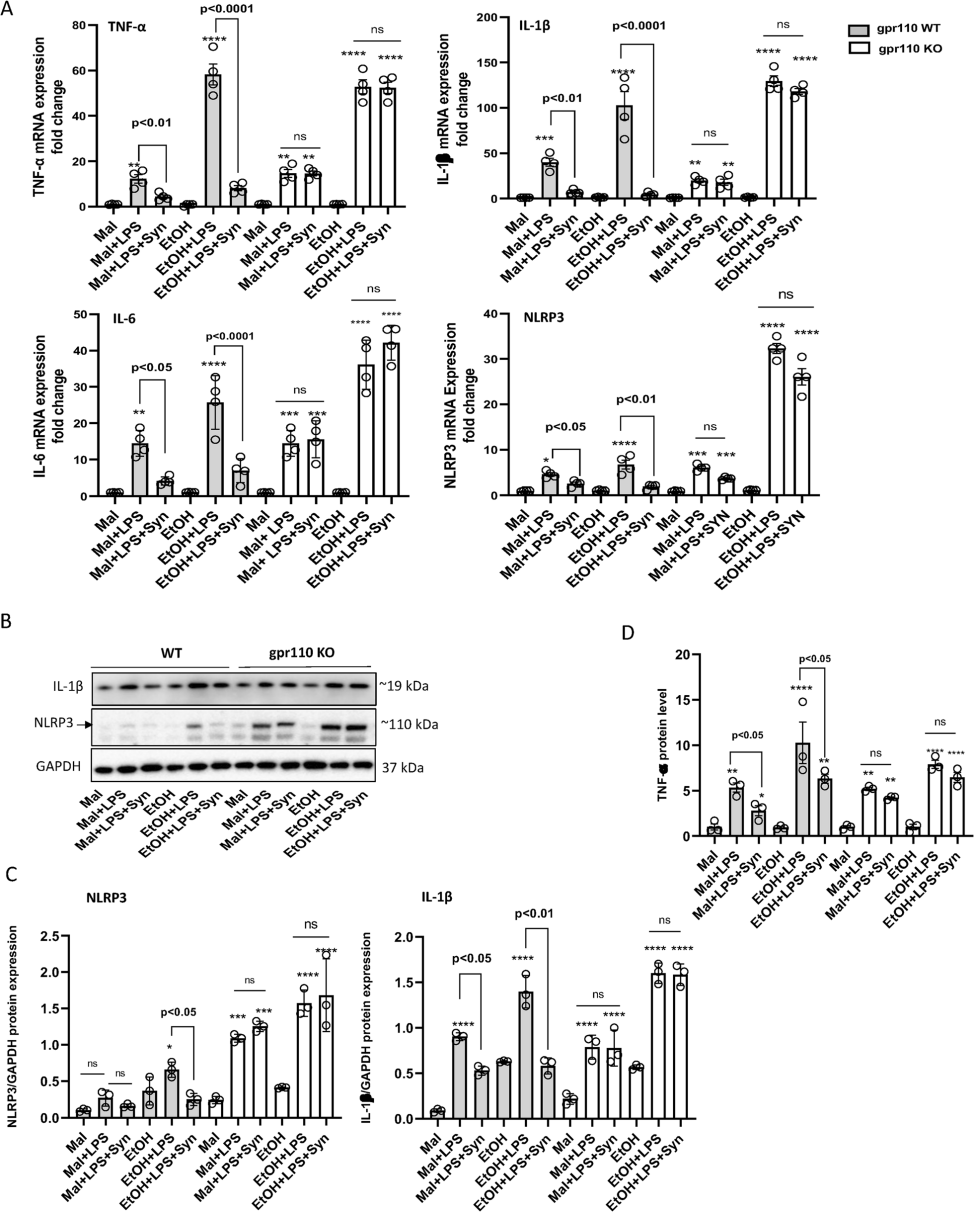
GPR110-dependent anti-inflammatory effects of synaptamide against LPS-induced neuroinflammation exacerbated by ethanol exposure. WT and GPR110 KO mice were administered with ethanol (3 g/kg) or maltose (5.4 g/kg) through oral gavage and LPS (1 mg/kg, i.p.) was injected at 4 h after ethanol administration. Synaptamide (5 mg/kg, i.p.) was injected immediately after LPS/synaptamide administration. The mRNA expression of TNF-α, IL-1β, IL-6 and nlrp3 (**A**) and the protein level of NLRP3 and IL-1β (**B**, **C**) were determined at 2 or 24 h after LPS injection, respectively. The cytokine level of TNF-α in blood was determined by ELISA at 2 h after LPS/synaptamide injection (**D**). All the values are presented as mean ± SEM (*n* = 4 for A; *n* = 3 for **B**–**D**), representing two independent experiments. ns, the difference of means is not statistically significant. *p < 0.05; **p < 0.01; ***p < 0.001; ****p < 0.0001 vs. Maltose group

Synaptamide has been previously shown to suppress LPS-induced neuroinflammation through activation of GPR110 [21]. To investigate whether GPR110 activation can suppress the exacerbating effect of ethanol in LPS-induced neuroinflammation, the synaptamide effect on the expression of proinflammatory cytokines was evaluated in WT and GPR110 KO mice (Fig. 2). Animals were administered with 3 g/kg ethanol through oral gavage, and 4 h later injected with LPS (1 mg/kg, i.p.) followed by synaptamide (5 mg/kg, i.p.). The proinflammatory mediators upregulated by LPS were further increased by ethanol pretreatment (Fig. 2). Compared to the maltose group (Mal + LPS), the LPS-induced mRNA expression was significantly elevated in ethanol-treated group (EtOH + LPS) for TNF-α, IL-6 and IL-1β in the WT brain (Fig. 2A; Table 1). Similar elevation was observed in KO animals for these cytokines when the EtOH + LPS group was compared to the Mal + LPS group. Subsequent injection of synaptamide significantly reduced the mRNA expression of these cytokines upregulated by ethanol and LPS by 70–90% in WT animals. However, no decrease in the mRNA expression of pro-inflammatory cytokines induced by LPS with or without ethanol-pretreatment was observed after administration of synaptamide in GPR110 KO animals. The single-dose ethanol exposure also upregulated the mRNA expression of NLRP3, an inflammasome component that is of critical importance for the innate immune system regulation [23], particularly in GPR110 KO animals. As observed with other proinflammatory cytokines, subsequent injection of synaptamide suppressed the LPS-induced increase in NLRP3 expression in a GPR110-dependent manner, suggesting a significant role of GPR110 in regulating NLRP3 expression in response to ethanol and LPS (Fig. 2A).

**Table 1:** Effect of ethanol on LPS-induced mRNA expression of proinflammatory cytokines in brain

Wild type				GPR110 KO		
LPS-induced fold change^a^				LPS-induced fold change		
Cytokine	Maltose	Ethanol	*p*-value^b^	Maltose	Ethanol	*p*-value
TNF-α	12.21 ± 3.66	58.25 ± 9.23	3.33E−02	14.64 ± 3.50	52.86 ± 6.24	3.98E−05
IL-6	14.46 ± 3.56	25.71 ± 7.36	3.33E−02	14.47 ± 3.56	36.11 ± 6.78	1.31E−03
IL-1β	40.14 ± 8.74	102.57 ± 26.74	8.08E−03	19.75 ± 4.10	129.58 ± 11.73	2.11E−06
NLRP3	4.80 ± 0.69	7.48 ± 1.51	8.10E−02	6.70 ± 0.85	31.85 ± 2.70	1.92E−06
AC8	0.51 ± 0.05	0.21 ± 0.02	7.41E−05	0.51 ± 0.10	0.24 ± 0.06	5.76E−03
PDE4B	1.77 ± 0.29	2.47 ± 0.24	2.70E−02	2.22 ± 0.34	4.82 ± 0.56	1.51E−04

^a^Relative to maltose control without LPS injection

^b^Calculated by Student’s *t*-test for fold changes of Ethanol + LPS vs. Maltose + LPS (*n* = 4)

A similar trend was observed with the protein level in that both ethanol and GPR110 activation influence the production of LPS-induced proinflammatory mediators (Fig. 2B, C). The LPS injection significantly upregulated NLRP3 and the cleaved form of active IL-1β, in both WT and GPR110KO mouse brains. The exacerbating effect of ethanol pretreatment observed with LPS-induced mRNA expression was also evident for these two proteins (Table 2). The LPS-induced production of IL-1β protein responded to ethanol pretreatment similarly in WT and KO brains. However, LPS-induced NLRP3 protein expression was particularly upregulated in GPR110 KO mouse brains regardless of ethanol pretreatment as observed from mRNA expression. Synaptamide significantly reduced the protein level of NLRP3 and IL-1β in WT but not in GPR110 KO brains, indicating a regulatory role of GPR110 activation in controlling not only the NLRP3 gene and protein expression but also the IL-1β protein production, an output of the NLRP3 inflammasome activation.

**Table 2:** Effect of ethanol on LPS-induced expression of proinflammatory proteins in brain

Wild type				GPR110 KO		
POI/GAPDH				POI/GAPDH		
Protein	Maltose	Ethanol	*p*-value^a^	Maltose	Ethanol	*p*-value
NLRP3	0.27 ± 0.11	0.67 ± 0.10	1.02E−02	1.09 ± 0.05	1.53 ± 0.15	9.00E−04
IL-1β	0.90 ± 0.04	1.39 ± 0.18	1.00E−02	0.78 ± 0.13	1.60 ± 0.11	1.00E−03
Iba-1	0.97 ± 0.01	2.18 ± 0.07	7.86E−06	1.37 ± 0.17	1.97 ± 0.45	1.00E−01
PDE4B	1.06 ± 0.07	1.40 ± 0.20	5.00E−02	1.00 ± 0.10	1.40 ± 0.26	7.00E−02

*POI* protein of interest

^a^Calculated by Student’s *t*-test for Ethanol + LPS vs. Maltose + LPS group (*n* = 3)

Single ethanol gavage given at 4 h prior to the LPS injection also elevated proinflammatory cytokines in the plasma collected at 2 h after LPS injection (Fig. 2D), indicating that LPS-induced systemic inflammation is also exacerbated by ethanol. Synaptamide treatment reduced the plasma protein level of TNF-α elevated by LPS injection alone or with ethanol gavage in WT animals. This reduction of pro-inflammatory cytokines in blood was not observed in GPR110 KO animals, indicating that suppression of LPS-induced and ethanol-enhanced systemic inflammation by synaptamide was mediated through GPR110 activation.

### LPS-induced and ethanol-exacerbated microglia activation is ameliorated by synaptamide-induced GPR110 activation

**Fig. 3:**
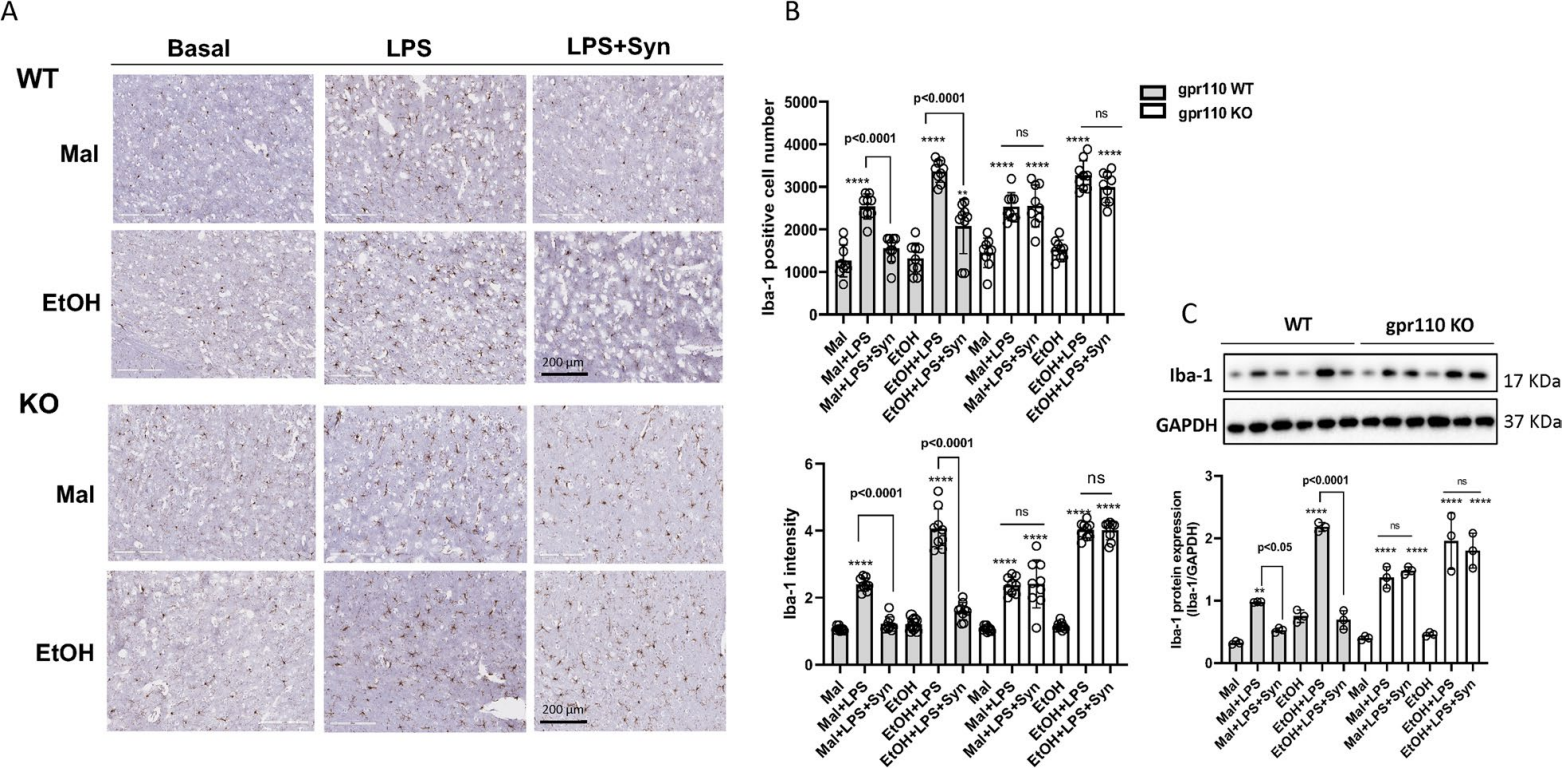
Suppression of LPS-induced and ethanol-exacerbated microglia activation by synaptamide-induced GPR110 activation. WT and GPR110 KO mice were administered with ethanol (3 g/kg) or maltose (5.4 g/kg) through oral gavage and LPS (1 mg/kg, i.p.) was injected at 4 h after ethanol administration. Synaptamide (5 mg/kg, i.p.) was injected immediately after LPS administration. Microscopic images were obtained from brain sections prepared at 24 h after treatment with LPS/synaptamide and immune-stained for Iba-1 (**A**). Iba-1 cell number and Iba-1 intensity was quantified (**B**). The Iba-1 protein level was determined by Western blot analysis (**C**) at 24 h after LPS/synaptamide injection. Values are presented as mean ± SEM (*n* = 3), representing two independent experiments. For microscopic data, 3 sections per each brain were quantified. ns, the difference of means is not statistically significant. **p < 0.01; ****p < 0.0001 vs. Maltose group

Microglia are the resident immune cells of the neurophysiological system that modulate the inflammatory responses in the brain. Systemic administration of LPS is known to activate microglia and production of pro-inflammatory cytokines in the brain, mediating neuroinflammation [20, 21, 24]. The effect of ethanol and GPR110 activation on the expression of Iba-1 in the brain cortex was examined at 24 h after LPS and synaptamide administration by immunohistochemistry and western blotting (Fig. 3; Additional file 1: Fig. S1). The Iba-1-positive cell number as well as Iba-1 intensity was significantly increased by LPS and further elevated by the pretreatment with ethanol in both WT and GPR110 KO mice (Fig. 3A, B; Additional file 1: Fig. S1). Synaptamide significantly reduced the Iba-1-positive cell number and Iba-1 intensity in WT but not in GPR110 KO brains. The western blot data from brain cortex also indicated that Iba-1 upregulation by LPS was further increased by single ethanol gavage given 4 h prior to LPS injection (Fig. 3C). Synaptamide reduced the Iba-1 protein level in WT but not in GPR110KO mice. These data consistently indicated the exacerbating effects of ethanol on microglia activation and ameliorating role of GPR110 activation in LPS-induced neuroinflammation.

### LPS-induced inflammatory responses are enhanced by ethanol and suppressed by synaptamide in cultured innate immune cells

**Fig. 4:**
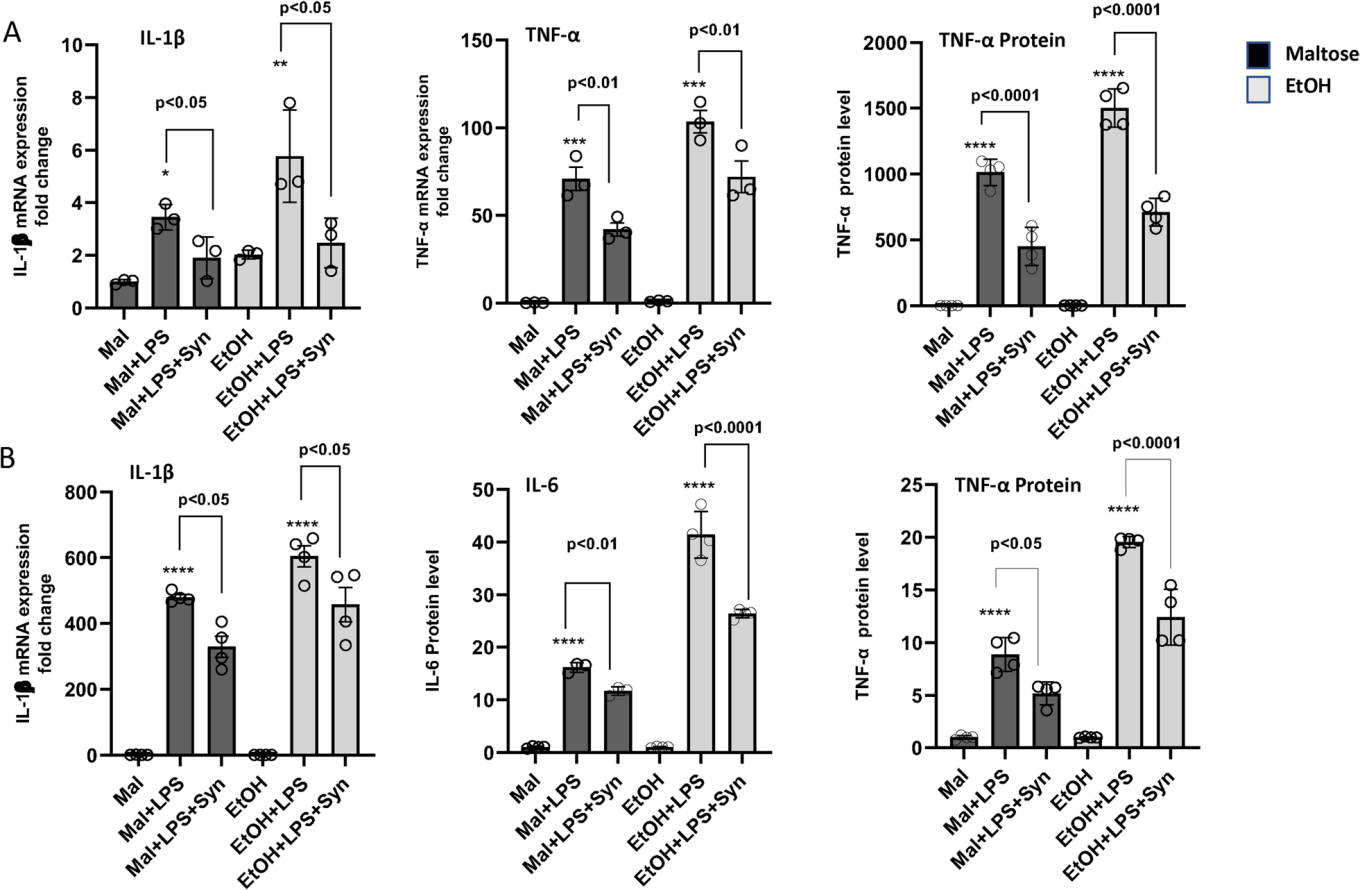
Ethanol-mediated potentiation of LPS-induced inflammatory responses and ameliorating effect of synaptamide in microglia and peritoneal macrophages in culture. Microglia and peritoneal macrophages were isolated from adult mice, incubated with 25 mM ethanol for 4 h and treated with LPS (100 ng/ml) for 1 or 12 h for mRNA or protein analysis, respectively. Synaptamide (10 nM) was added to the cell culture immediately after LPS treatment. The mRNA expression of IL-1β, TNF-α or IL-6 and TNF-α protein level were measured in microglia (**A**) and peritoneal macrophages (**B**) and the data are presented as the fold change relative to the maltose control (MAL). Ethanol potentiated the LPS-induced proinflammatory cytokine expression at both mRNA and protein levels while synaptamide suppressed the effect of LPS and ethanol in microglia and macrophage cells in culture. Values are presented as mean ± SEM (*n* = 3–4), representing two independent experiments. ns, the difference of means is not statistically significant. *p < 0.05; **p < 0.01; ***, *p* < 0.001; ****p < 0.0001 vs. Maltose group

Single treatment with ethanol followed by LPS injection increased the proinflammatory mediator level in brain and plasma, and synaptamide reduced their level (Figs. 2B–D, 3). Since innate immune cells both in the brain and periphery are activated by LPS for inflammatory responses, we also examined the exacerbating effect of ethanol in vitro in cultured microglia and peritoneal macrophages where GPR110 expression has been demonstrated [21] (Fig. 4). Pretreatment of these innate immune cells with ethanol for 4 h prior to LPS addition (100 ng/ml) resulted in further increases in LPS-induced mRNA expression of IL-1β and TNF-α in microglia and IL-1β in macrophages. The presence of ethanol increased the secretion of TNF-α into the medium in microglia or IL-6 and TNF-α secretion in peritoneal macrophages by 1.5- to 2.5-fold compared to LPS treatment alone. Treatment with 10 nM synaptamide following LPS addition significantly downregulated the induction of IL-1β, TNF-α and IL-6 caused by LPS and LPS + EtOH in macrophages and microglia in vitro.

### In vivo anti-inflammatory action of GPR110 is mediated through modulation of the cAMP system in LPS-induced and ethanol-enhanced neuroinflammation

**Fig. 5:**
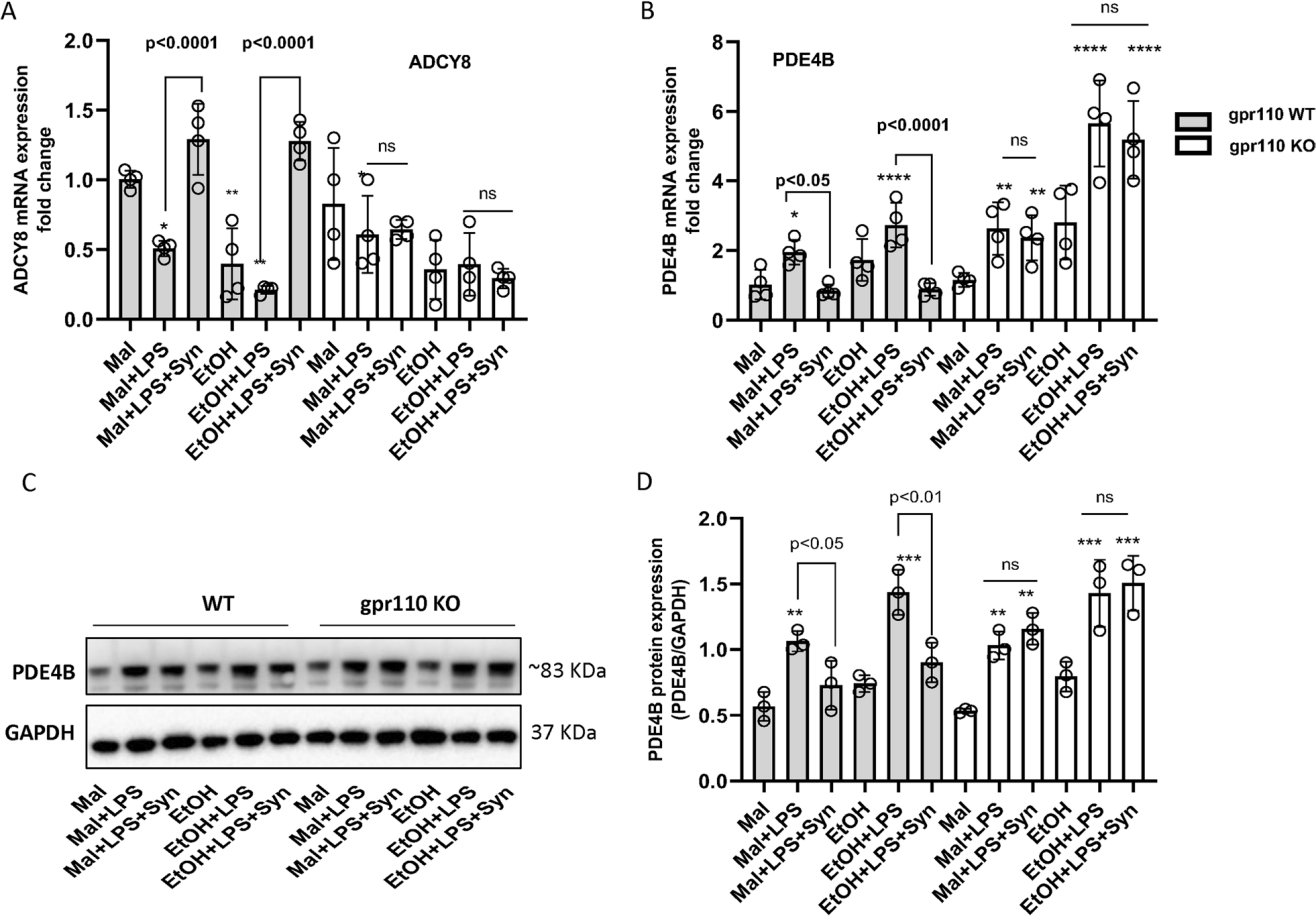
Opposing effects of ethanol and synaptamide on the cAMP system in LPS-induced neuro-inflammation. WT and GPR110 KO mice were given 3 g/kg ethanol through oral gavage and LPS (1 mg/kg, i.p.) was injected at 4 h after ethanol administration. Synaptamide (5 mg/kg, i.p.) was injected immediately after LPS administration. The expression of mRNA and protein in brain tissues was measured for isoforms of AC (ADCY) and pde4 at 2 and 24 h after LPS injection, respectively. The mRNA expression of ADCY8 (**A**) and PDE4B (**B**) were perturbed by LPS which was potentiated by ethanol. Synaptamide GPR110-dependently restored the reduced expression of ADCY8 caused by LPS and EtOH + LPS and reduced the PDE4B expression elevated by LPS and EtOH + LPS. The western blot analysis (**C**, **D**) showed an LPS-induced increase in PDE4B protein which was further elevated by ethanol pretreatment but was suppressed by synaptamide in a GPR110-dependent manner. Values are presented as mean ± SEM (*n* = 4 for **A**, **B**; *n* = 3 for **C**, **D**), representing two independent experiments. ns, the difference of means is not statistically significant. *p < 0.05; **p < 0.01; ***p < 0.001; ****p < 0.0001 vs. Maltose group

Perturbation of the cAMP system has been considered an integral part of as well as underlying mechanism for ethanol-induced pathophysiology [25]. It also has been reported that synaptamide upregulates the cAMP system by activating GPR110 [20]. As adenylyl cyclase (AC) and cAMP-specific phosphodiesterase 4 (PDE4) are responsible for the synthesis and breakdown of cAMP, we examined the effect of single dose of ethanol on the expression of AC and PDE4 isoforms in the mouse brain after LPS injection (Fig. 5; Additional file 1: Fig. S2). The mRNA expression of a specific AC isoform AC8 (ADCY8) in the WT brain was significantly reduced by 50–60% after either LPS administration or single-dose ethanol exposure (Fig. 5A; Table 1) while other AC isoforms were not affected significantly (Additional file 1: Fig. S2A). The ADCY8 mRNA expression was reduced by LPS and was further downregulated (by 80%) in the presence of ethanol compared to the maltose control. AC8 expression in GPR110 KO brains also showed a similar trend although the difference was not statistically significant. Synaptamide restored the reduced expression of ADCY8 caused by LPS alone or together with ethanol pretreatment in WT, but it exerted no effect in GPR110 KO mice. The mRNA expression of the isoform AC4 (ADCY4) also showed a similar response to LPS and synaptamide although statistical significance was not reached in most cases, but an effect of ethanol was not observed (Additional file 1: Fig. S2A).

In contrast to AC8, intraperitoneal administration of LPS or single-dose ethanol exposure increased the mRNA expression of a particular isoform PDE4B by 1.77 ± 0.29 fold in WT and by 2.47 ± 0.24 fold in GPR110 KO mouse brains compared to the maltose control (Fig. 5B; Table 1). Further upregulation of PDE4B mRNA was observed when LPS was injected after ethanol pretreatment (2.47 ± 0.24 vs. 1.77 ± 0.29, *p* = 2.70E−02 for WT), particularly in GPR110 KO where the expression of PDE4B mRNA was prominently upregulated (4.82 ± 0.56 vs. 2.22 ± 0.34, *p* = 1.51E−04 for KO). Treatment with synaptamide significantly blocked the LPS-induced increase of PDE4B mRNA expression in the WT but not in GPR110 KO mice. Besides PDE4B, no significant changes in the mRNA expression were observed for other isoforms of pde4 in response to LPS or ethanol (Additional file 1: Fig. S2B). Western blot analysis similarly indicated that the protein level of PDE4B in the brain increased significantly after LPS injection with or without ethanol pretreatment, and ethanol pretreatment potentiated the LPS-induced increase in PDE4B protein level (Fig. 5C, D; Table 2). The observed upregulation of PDE4B was suppressed by synaptamide in WT but not in GPR110 KO mice. These results indicated that both ethanol and LPS modulated the cAMP system at least in part by downregulating AC8 and upregulating PDE4b isoform, and synaptamide GPR110-dependently offset the effects of ethanol and LPS on AC8 and PDE4B at the transcript level, and also at the protein level in case of PDE4B.

## Discussion

In this study, we demonstrated the exacerbating effect of single ethanol exposure on LPS-induced neuroinflammation through elevating immune responses in the brain and in the circulation. GPR110 activation by its ligand synaptamide ameliorated the LPS-induced and ethanol-exacerbated proinflammatory responses through counter-acting on the cAMP system and NLRP3 inflammasome, revealing potential targets for ethanol and synaptamide interaction in neuroinflammation.

Exposure to ethanol in various experimental paradigms has been shown to produce immune responses in the central nervous system. For example, binge ethanol drinking of 2 or 3 g/kg for 3 times per day for 2 consecutive days followed by 5 days of abstinence resulted in significant increase in activated microglia in the hippocampal dentate gyrus area of the rat brain [26]. Chronic ethanol treatment for about a month followed by a single acute binge significantly upregulated mRNA expression of pro-inflammatory mediators such as IL-1β, TNF-α, CCL2 and COX-2 and inflammasome components NLRP3 and caspase-1 in mouse hippocampus and cerebellum [27]. Likewise, after repetitive binge ethanol intoxication, elevation of neuro-inflammation-linked proteins in rat hippocampus and entorhinal cortex has been reported [28]. Single-dose ethanol exposure was also shown to produce lasting changes in synaptic morphology and mitochondrial trafficking in mice [29] and to elevate IL-6 and IκBα expression in rat brain regions [30]. Our study demonstrates that even one-time ethanol gavage at 3 g/kg which acutely increased the blood alcohol concentration can potentiate neuroinflammatory responses induced by systemic LPS injection in mice.

According to our earlier studies, systemic administration of LPS or injury can upregulate GPR110 in the brain, and administration of GPR110 ligands ameliorates inflammatory responses through the cAMP/PKA/CREB singling pathway in vivo and in cultured microglia [20–22]. Recent studies also reported that synaptamide reduced neuroinflammation and cognitive or visual impairment in animal models of traumatic brain injury [22] and ethanol exposure is involved in neuroinflammation [31–33]. In the present study, single-dose ethanol exposure was found to elevate the GPR110 expression and this increase was further potentiated by LPS injection (Fig. 1C). Apparently, elevated GPR110 expression contributed to the effective suppression of inflammatory responses by synaptamide in the current paradigm of LPS-induced neuroinflammation exacerbated by single-dose ethanol exposure (Figs. 2, 3).

Microglia are the CNS immune cells that respond to inflammatory signals involved in both acute and chronic ethanol exposure [31–35]. Upregulation of Iba-1 expression and morphological alteration of microglia in the cortex and hippocampus of mice have been observed after chronic and acute ethanol feeding [36]. The present study also indicates that single-dose ethanol gavage potentiated Iba-1 expression increased by LPS in the brain (Fig. 3; Additional file 1: Fig. S1).

We found that LPS-induced expression of NLRP3, an important inflammasome component, is potentiated by ethanol, and is particularly exaggerated in the absence of GPR110 (Fig. 2). The NLRP3 is a multiprotein complex assembly that regulates inflammatory responses and cellular stress through cleavage of the cytokine IL-1β with the help of caspase1 [37, 38]. Activation of NLRP3 inflammasome requires initial priming to help the transcriptional upregulation of the inflammasome complex components [39]. This transcriptional upregulation during priming has been shown to be mediated through activation of toll-like receptors, IL-1 receptors and tumor necrosis factor receptor that ultimately activate NF-κβ for transcription [40]. It has been previously reported that chronic ethanol treatment can amplify IL-1β secretion upon treatment with NLRP3 agonists in human peripheral blood mononuclear cells and a mouse macrophage cell line [41]. It also has been documented that NLRP3 deletion can protect against alcohol-associated increases in caspase-1 and IL-1β levels in the mouse brain [42]. Single-dose acute exposure to ethanol employed in our study also resulted in the elevation of LPS-induced NLRP3 expression and increases in IL-1β production. These findings suggest that the NLRP3 inflammasome is a synergistic target of ethanol for inflammatory responses. The prominent upregulation of LPS-induced NLRP3 transcription in ethanol-treated GPR110 KO mice observed in the current study conversely indicates a significant regulatory role of GPR110 in initial priming of the NLRP3 inflammasome in response to LPS-induced TLR activation.

It has been reported that cAMP suppresses NLRP3 inflammasome activation by directly binding to the nucleotide binding domain, and NLRP3 activation can be reduced by inhibiting cAMP degradation [43]. It has been reported that the cAMP system is a shared target for ethanol and synaptamide [20, 44] which may provide an explanation for immune-regulatory effects of synaptamide on ethanol-exacerbated inflammatory responses. Decline in cAMP through PDE4B upregulation was shown to play a key role in the activation of glial cells and neuroinflammation induced by chronic ethanol [45]. Earlier it also has been reported that chronic ethanol downregulates AC8 expression thereby reducing cAMP level in mouse neural stem cells [44]. Synaptamide not only upregulated AC8 level and promoted neurogenesis in the presence of ethanol but also acts as priming agent for AC8 induction and cAMP production to restore impaired neurogenesis in vitro*.* Moreover, synaptamide has been shown to suppress LPS-induced inflammatory responses in a GPR110/cAMP-dependent manner in innate immune cells. Our current study reveals that LPS and acute ethanol exposure significantly downregulate the cAMP system by perturbing AC8 and PDE4B expression, while synaptamide-induced GPR110 activation affects these targets in an opposite direction (Fig. 5). The anti-inflammatory function of GPR110 may be mediated at least in part through offsetting the effects of ethanol and LPS on AC8 and PDE4B.

**Fig. 6:**
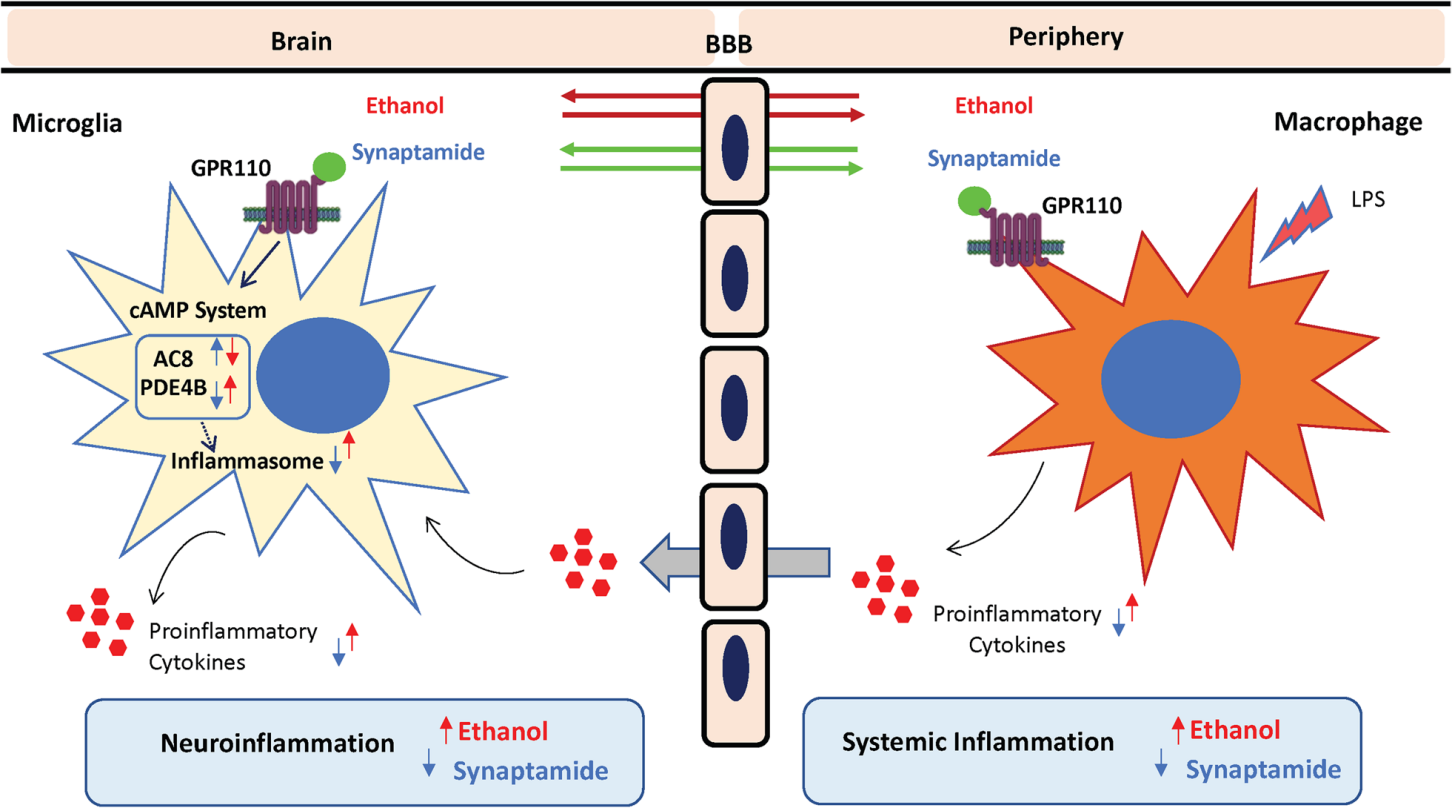
Schematic representation of the proposed model for immune regulatory function of synaptamide-induced GPR110 activation in LPS-induced neuroinflammation exacerbated by ethanol. LPS activates peripheral immune cells such as macrophages and increases the level of pro-inflammatory mediators. Priming with ethanol exacerbates inflammatory responses induced by LPS. The cytokines produced by peripheral immune cells cross the blood–brain barrier and activate microglia, resulting in neuroinflammation through downregulating the cAMP system and activating NLRP3 inflammasome. Pharmacological administration of synaptamide exerts its therapeutic effect on central and peripheral targets that express GPR110 or induce GPR110 expression after LPS and/or ethanol challenge. By activating GPR110, synaptamide ameliorates neuroinflammation under ethanol exacerbated conditions by upregulating the cAMP production system and suppressing NLRP3 inflammasome activation. Ethanol and synaptamide can cross the blood–brain barrier

In addition to microglia, peripheral immune cells play a crucial role in producing LPS-induced neuroinflammatory responses [21, 46]. The LPS-induced inflammatory responses were enhanced by ethanol and suppressed by synaptamide in microglia and macrophages (Fig. 4), suggesting that both brain and peripheral immune cells contribute to the observed neuroinflammatory modulation caused by these agents in vivo as depicted in Fig. 6. Ethanol priming increases LPS-induced production of pro-inflammatory cytokines in macrophages. Increased pro-inflammatory cytokines in the systemic circulation traverse BBB and potentiate microglial activation and inflammatory responses through downregulating the cAMP system and activating NLRP3 inflammasomes. Synaptamide, by activating GPR110 in both peripheral and central immune cells, upregulates the cAMP system, counteracts the effect of LPS and ethanol on NLRP3 inflammasomes, and suppresses LPS-induced, ethanol-exacerbated neuroinflammation. The anti-inflammatory effect of synaptamide through activation of the GPR110 receptor in vivo might prove to be of therapeutic use for early stages of neurodegenerative conditions associated with neuroinflammation.

## Conclusion

We have demonstrated for the first time that single-dose ethanol exposure for as little as 4 h exacerbates neuroinflammatory responses induced by systemic LPS administration through potentiating an LPS-induced perturbation of the cAMP system, specifically AC8 and PDE4B, and upregulating NLRP3 inflammasome. Anti-inflammatory effects exerted by GPR110 activation is mediated through counteracting on AC8 and PDE4 and suppressing NLRP3 inflammasome, revealing the cAMP system and NLRP3 inflammasome as common targets for ethanol and GPR110 signaling. GPR110 activation by its ligands may have therapeutic potential for neuroinflammation aggravated by ethanol consumption.

### Supplementary Information

**Additional file 1: Fig. S1. **Magnified images of microglia activated by LPS, exacerbated by pre-exposure to ethanol and attenuated by synaptamide-induced GPR110 activation. Fig. S2. Effect of LPS, ethanol and synaptamide on mRNA expression of adenylylcyclase (ADCY) and PDE4 isoforms in the mouse brain. WT mice (n = 4 for each group) were given 3 mg/kg ethanol through oral gavage and LPS (1 mg/kg, i.p.) was injected at 4 h after ethanol administration. Synaptamide (5 mg/kg, i.p.) was injected immediately after LPS administration. At 2 h after LPS injection, the mRNA expression of adenylylcyclase (A) and PDE4 isoforms (B) were measured. No significant effects were observed except for LPS-induced PDE4D expression where the elevation by ethanol (p < 0.05 vs. Maltose group) and prevention by synaptamide showed significant differences (p < 0.05 vs. EtOH + LPS group).

## Data Availability

Limited raw data and materials can be provided if needed.

## Abbreviations

AC: Adenylyl cyclase
BBB: Blood–brain barrier
CNS: Central nervous system
GPCR: G-protein coupled receptor
LPS: Lipopolysaccharide
NLRP3: NOD-like receptor family pyrin domain containing 3
PDE: Phosphodiesterase
Synaptamide: *N*-Docosahexaenoylethanolamine
