# Supplementary material for: Exacerbating effects of single-dose acute ethanol exposure on neuroinflammation and amelioration by GPR110 (ADGRF1) activation
Source: J Neuroinflammation. 2023 Aug 14;20:187. doi: 10.1186/s12974-023-02868-w (PMC10426059; doi:10.1186/s12974-023-02868-w)
Supplement: Supplementary file 1 — Additional file 1: Fig. S1. Magnified images of microglia activated by LPS, exacerbated by pre-exposure to ethanol and attenuated by synaptamide-induced GPR110 activation. Fig. S2. Effect of LPS, ethanol and synaptamide on mRNA expression of adenylylcyclase (ADCY) and PDE4 isoforms in the mouse brain. WT mice (n = 4 for each group) were given 3 mg/kg ethanol through oral gavage and LPS (1 mg/kg, i.p.) was injected at 4 h after ethanol administration. Synaptamide (5 mg/kg, i.p.) was injected immediately after LPS administration. At 2 h after LPS injection, the mRNA expression of adenylylcyclase (A) and PDE4 isoforms (B) were measured. No significant effects were observed except for LPS-induced PDE4D expression where the elevation by ethanol (p < 0.05 vs. Maltose group) and prevention by synaptamide showed significant differences (p < 0.05 vs. EtOH + LPS group). [file 12974_2023_2868_MOESM1_ESM.pdf]

Additional file 1. Fig. S1

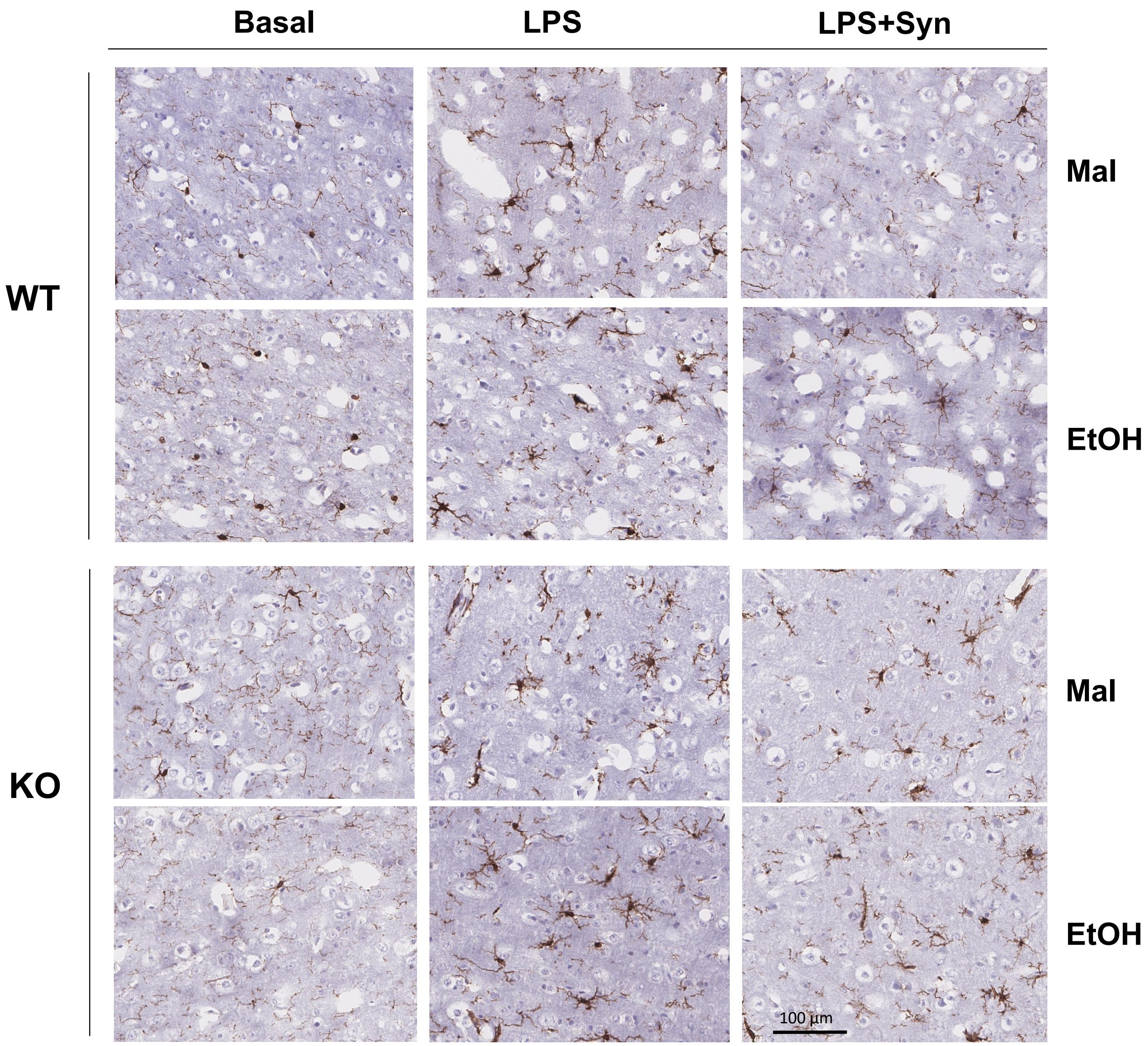

Additional file 1. Fig. S2

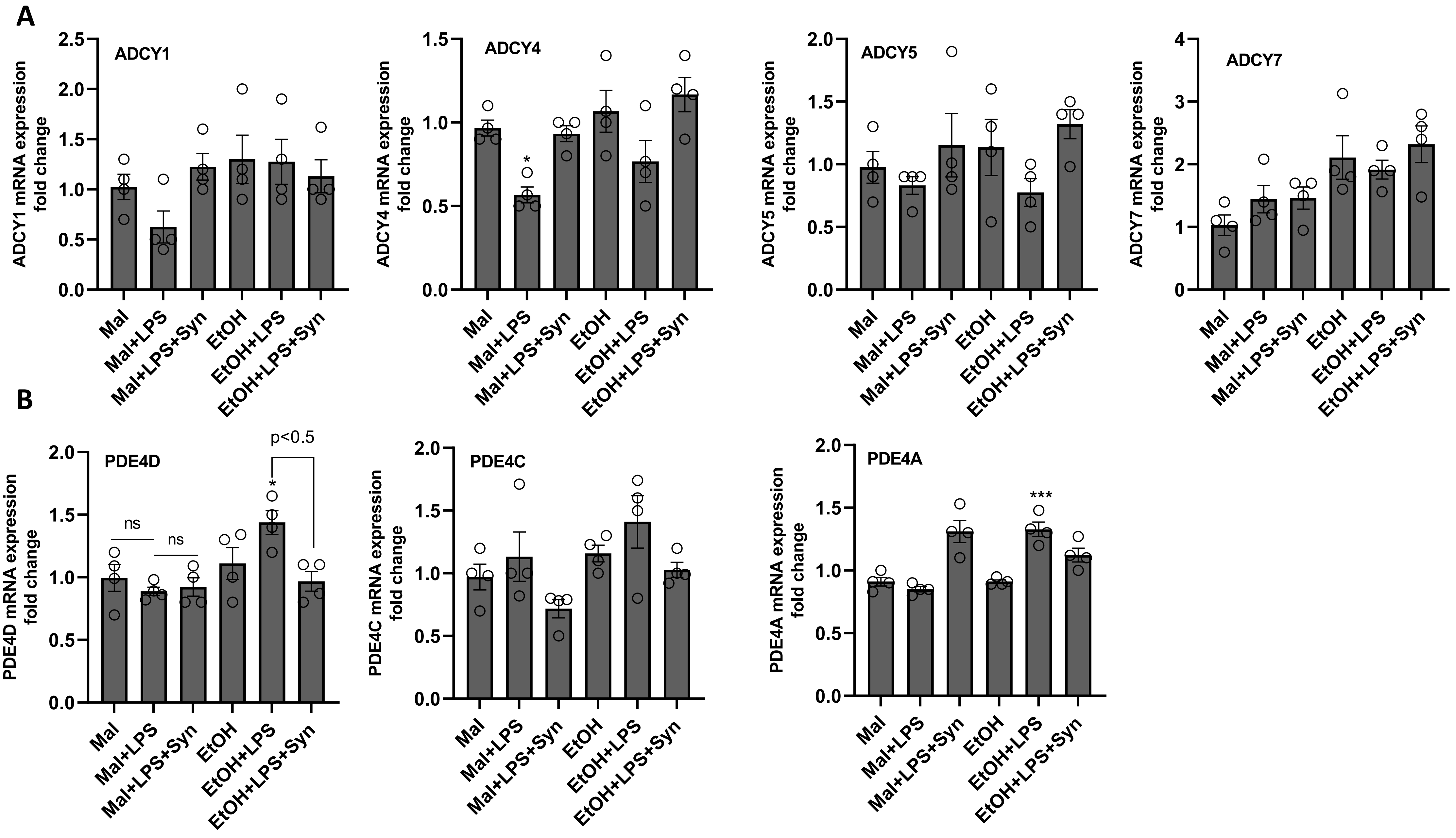

Additional file 1

Raw data for WB images

WT

1. Mal
2. Mal+LPS
3. Mal+LPS+Syn
4. EtOH
5. EtOH+LPS
6. EtOH+LPS+Syn

GPR110 KO

7. Mal
8.Mal+LPS
9.Mal+LPS+Syn
10.EtOH
11.EtOH+LPS
12.EtOH+LPS+Syn

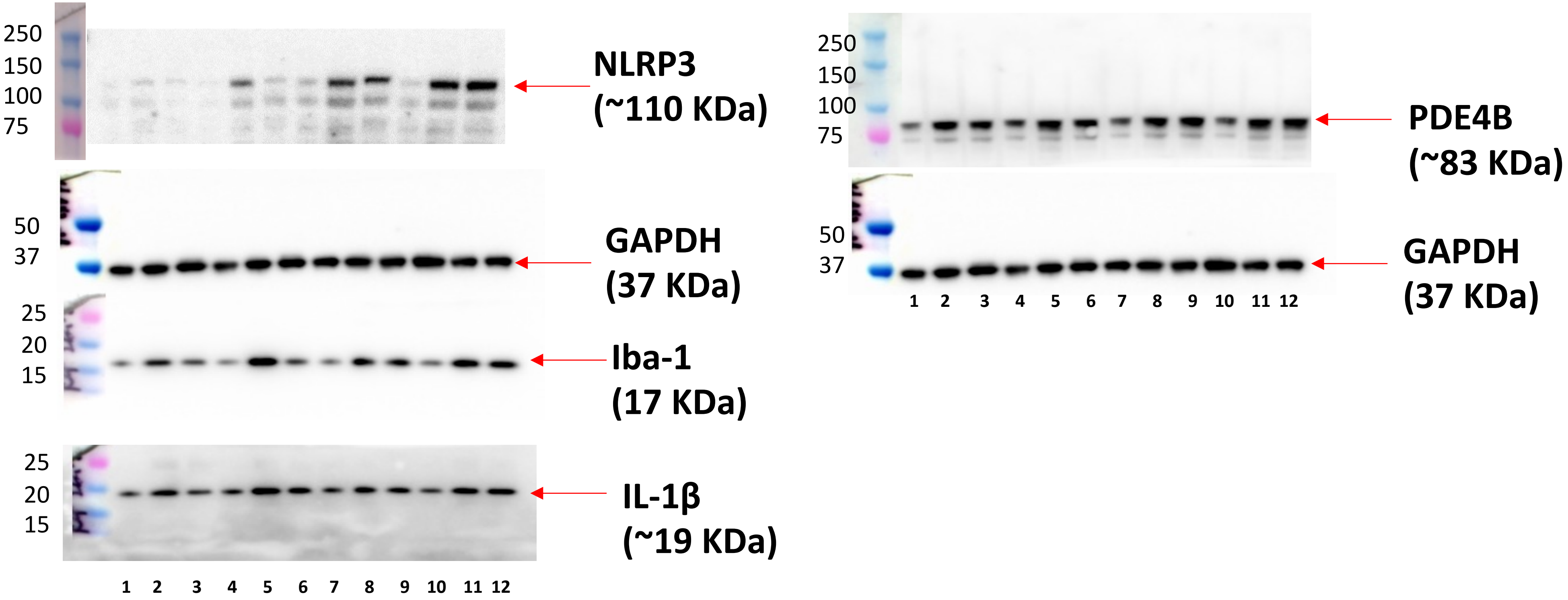

IL-1β was reprobed from Iba-1 membrane
